# Singular Effects of Snake Poison

**Published:** 1860-06

**Authors:** 


*Singular Effects of Snake Poison.*—We cull the following details of a somewhat singular case, from a private letter from Dr. Sweeney, of Rushville, Ill. It was laid aside for use in this way at the time of its reception, some months ago, but by accident mislaid. He writes: "Mr. DeCamp, of this county, was bit by a copper-head snake, in the State of Pennsylvania, thirty years ago; he was treated at the time by his neighbors; whiskey was the only remedy used. His health, from the time of his recovery from the effects of the bite, was pretty good till 1857—about the same time of the year he was bitten—when he broke the skin on the back of the hand that had been bitten so many years ago. Inflammation set in, with pain, uneasiness of the stomach, delirium, and spots over the general surface, the color of the snake.—Remedies were tried, without relief. It was suggested to him by a friend, on seeing the spots on the skin, to try the 'whiskey practice.' By its free use he was soon relieved of the general syptoms, but still experienced slight uneasiness in the whole arm; and particularly the middle fingers of the hand, which had originally received the injection of the snake poison, till the winter of 1858, when contraction of the fingers came on, with pain through the whole course of the arm, and to the chest. The general health became bad, but has since greatly improved. The fingers remain contracted, and the whole arm much weakened. I was approached by the family for advice, but I had none to give; and would be much obliged to have remedies suited to this case pointed out, through the *Lancet & Observer*."—*Lancet & Observer*.

